# Corrupted colonic crypt fission in carcinogen-treated rats

**DOI:** 10.1371/journal.pone.0172824

**Published:** 2017-03-08

**Authors:** Carlos A. Rubio

**Affiliations:** Department of Pathology, Karolinska Institute and University Hospital, Stockholm, Sweden; Rutgers, the State Univesity of New Jersey, UNITED STATES

## Abstract

**Background:**

The colonic crypts in rats reproduce themselves by symmetric fission at the base of the crypts, and proceeding upwards, generate two separate identical crypts. Recently we reported corrupted colonic crypt fission (CCCF) in rats with colonic carcinoma. Here we investigated whether CCCF also occurred in the colonic mucosa without carcinoma in carcinogen-treated rats.

**Methods:**

Filed Swiss-roll sections from 35 male rats (25 treated with 1,2-dimethyhydrazine (DMH) suspended in EDTA solution, and 10 EDTA-treated) were reviewed. CCCF were regarded those with either asymmetric basal fission, asymmetric lateral sprouting/lateral fission, basal dilatations, or spatial aberrations of the normal (vertical) axis.

**Results:**

202 CCCF (38%) were recorded amongst 533 crypts with fission in DMH-treated rats, and only one CCCF (0.1%) was found amongst 571 crypts with fission in EDTA-treated rats (p<0.05). The basal aspect of four adenomas included in Swiss roll sections exhibited CCCF lined either with indigenous (non-dysplastic) epithelium or with dysplastic epithelium.

**Conclusion:**

It was demonstrated that CCCF without dysplasia develop in carcinogen-treated SD rats. As judged by the figures presented, the possibility that the epithelium in those corrupted crypts was successively replaced by top-down growing dysplastic cells, could not be totally rejected. This is the first report showing that non-dysplastic CCCF may antedate the very early stages of colonic carcinogenesis in SD rats.

## Introduction

While attempting to produce amyotropic lateral sclerosis by feeding nuts of *Cycas circinalis* (a tropical fern from a family of *Cycadaceae*), Laqueur *et al*. accidentally found that rats had developed colonic tumours [1]. The same author subsequently demonstrated that the active carcinogen in these nuts was cycasin, a water-soluble á-glucoside of methylazoxymethano [2]. That discovery, lead Ducky *et al*. to administer a structurally similar compound, namely 1,2-dimethylhydrazine (DMH), to induce tumours in the colon of rats [3]. Since then, DMH and its carcinogenic metabolites (azoxymethane (AOM) and naethylazoxy methanol) have been commonly used to study morphology, pathogenesis, prevention and treatment in experimentally induced colonic tumours [4–6].

In both rodents and humans, most carcinomas evolve in the mucosal domain that occupies the vast majority of the colonic mucosa. This mucosal domain is built with crypts lined by goblet cells and columnar cells. Carcinomas in this vast domain are preceded by foci of dysplastic cell proliferations called conventional adenomas [7] or serrated adenomas [8]. Progression to carcinoma is triggered by the accumulation of molecular aberrations [9] and by epigenetic modification of gene expression [10]. The remnant mucosal domain, known as gut-associated lymphoid tissue (GALT) mucosa, is built with tiny organized lymphoid follicles [11]. Puzzlingly, colonic GALT carcinomas are frequent in carcinogen-treated rats [12] but rare in humans [13].

Few *in vivo* studies have been done to unveil a possible morphologic stage preceding crypt dysplasia and adenomas in rodents. In this context Bird *et al*. detected in the colonic mucosa of rodents treated with carcinogens, gross changes in the pit pattern consisting in groups of 5 or more colonic crypts [14]. These changes, called aberrant crypt foci (ACF), were regarded as potential markers of colorectal cancer risk as their incidence at an early time point highly correlated with end-point tumour incidence [14]. Subsequently Nascimbeni *et al*. found that ACFs occurred more frequently in patients with CRC than in controls, and claimed that the phenomenon sustained their putative role as a preneoplastic marker [15]. Nonetheless, the same group reported 10 years later that ACFs occurred less frequently in the distal colon of carcinogen-treated rats, which is the site of predilection for the development of adenomas and carcinomas. Their conclusion was that the growing features and allocation did not sustained the notion of ACF as a pre-neoplastic biomarker [16]. In another study, Ochiai *et al*. [17] submitted that the specificity of ACF as tumor precursors should be questioned, partly because of their predominant location in the proximal colon where tumors do not develop, and by their gradual disappearance over time [17]. Moreover, ACF frequently harboured Kras mutations, but rarely adenomatous polyposis coli (APC) mutations [17] strongly suggesting that the majority of ACF might not be true preneoplastic lesions.

Recently, while reviewing archival colonic sections from previous experiments in rats [18, 19], we noticed CCCF on top of GALT-carcinomas [20]. These observations prompted us to investigate whether similar colonic crypt alterations could occur in the vast GALT-free mucosal domain in carcinogen-treated rats.

## Materials & methods

Sprague-Dawley rats (Anticimex, Stockholm, Sweden) weighing 200 g were used. The animals were kept 5 rats/cage and were fed with a purine chow diet (R3; Ewos, Astra, Södertälje, Sweden). All rats were injected s.c. with 21 mg/kg of body weight of 1,2-dimethylhydrazine (DMH) hydrochloride salt (MW, 133–02) (Kebo, Stockholm, Sweden) suspended in 1 ml of EDTA solution (as a stabilizing agent) once a week for 27 weeks. The animals were weighted once in 2 weeks and the DMH dose adjusted accordingly.

Filed histological Swiss-roll sections from 35 male Sprague-Dawley (SD) were reviewed. Out of the 35 Sprague-Dawley rats, 25 were injected subcutaneously with a weekly dose of 21 mg/kg body weight of 1,2-dimethylhydrazine (DMH), hydrochloride salt Nw 133.02, for 27 weeks [18], and the remaining 10 SD rats were injected with EDTA for 27 weeks until sacrifice. The Swiss-roll technique was performed in 40 rats without visible tumours and in four rats showing small polyps.

Quantification of crypts with symmetric and asymmetric fission was performed on entire Swiss-roll sections.

### Statistical analysis

The non-parametric Mann-Whitney test was applied, to compare difference between groups. Statistical significance was defined as *P*<0.05.

The Ethical Committee of the Karolinska Institute approved the old experiments in Sweden (N 48/1989).

## Results

### Normal colonic crypts

**Fig 1:**
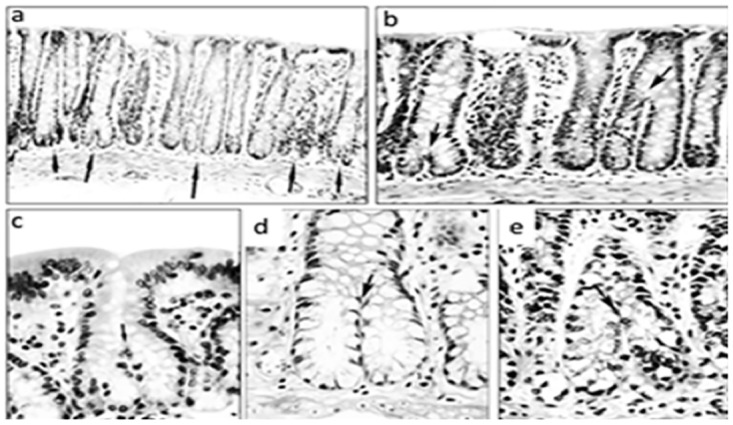
Various stages of normal symmetric colonic crypt fission at arrows (EDTA-treated rats, hematoxylin-eosin, a: x4, b:x10, c, d, e: x20).

The normal colon of untreated rats is built with test tube-shaped, close-packed crypts with their axis vertical to the *muscularis mucosae*. The colonic crypts reproduce themselves by symmetric fission, the division of which begins at the base of the crypts and proceeds upwards until they result into two identical, individual crypts [21] (Fig 1).

The crypts in the distal colon are easily recognized since they exhibit increased numbers of mucus-producing goblet cells in their lower segment. Mucus production is essential for lubricating dehydrated faeces on their way to the rectal ampullae.

### Corrupted Colonic Crypt Fission (CCCF)

**Fig 2:**
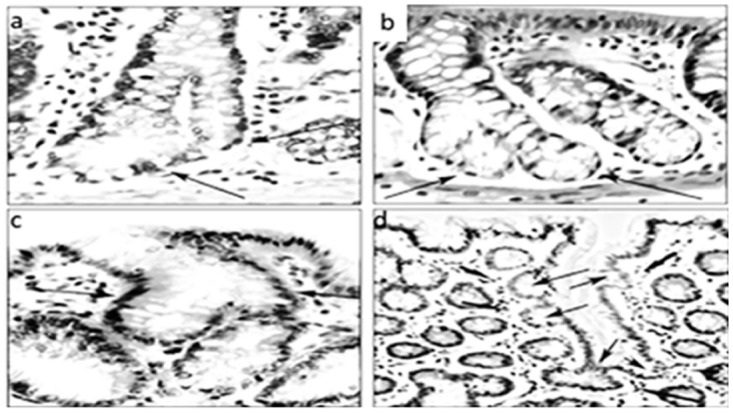
Corrupted colonic crypts showing asymmetric fission (a, b and c) and asymmetric lateral sprouting (d) at arrows (DMH-treated rats, hematoxylin-eosin, a, b and c: x20, d: x10).

**Fig 3:**
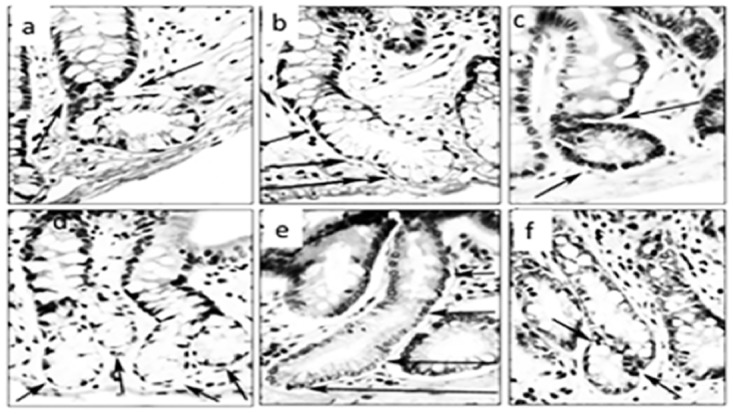
Corrupted colonic crypts exhibiting spatial aberrations of the normal (vertical) axis at arrows. Note L-shaped crypts (**a** and **c**), crypts with lateral sprouting (**d** and **f**), and with oblique axis (**b** and **e**) (DMH-treated rats, hematoxylin-eosin, **a** to **f**: x20).

**Fig 4:**
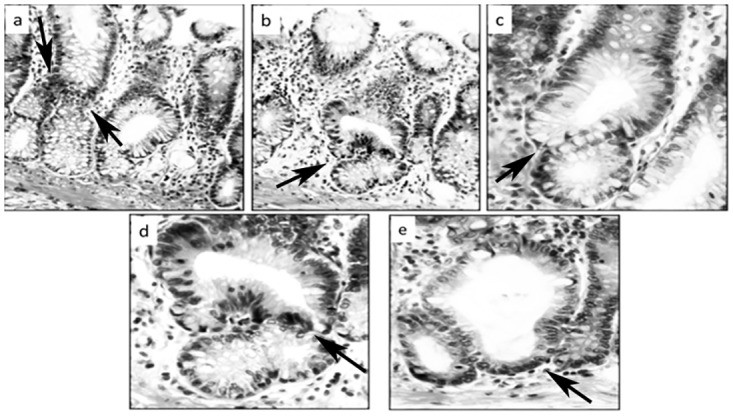
Corrupted colonic crypts showing various degrees of corrupted fission at arrows, some with abnormal basal crypt dilatation. Note low-grade dysplasia (**b** to **e**; **d**: detail of figure **b**). (DMH-treated rats, hematoxylin-eosin, **a**, **b** x20, **c, d, e** x40).

As CCC were regarded those with: *i)* Asymmetric basal fission, *ii)* Asymmetric lateral fission, *iii)* Asymmetric lateral sprouting, *iv)* Crypts with basal dilatation (≥ than twice the diameter of the normal lumen), and *v)* Crypts with spatial aberrations of the normal (vertical) axis (Figs 2–4)

#### I) Frequency of CCCF in DMH-treated, and EDTA-treated rats

Table 1 shows that 202 CCCF were recorded in the 25 DMH-treated rats (8.08 CCCF/DMH-treated rat, range 26–1), and one CCCF among the 10 EDTA-treated rats (0.1 CCCF/ rat, range 1–0). The difference of CCCF between DMH-treated rats and EDTA-treated rats was significant (p<0.05).

**Table 1:** The number of colonic crypts with symmetric fission and with corrupted fission in 35 male rats: 25 Sprague-Dawley (SD) rats injected for 27 weeks with 1,2 Dimethylhydrazine (DMH) suspended in EDTA, and 10 male SD rats injected with EDTA for 27 weeks.

	Symetric crypts DMH	Symetric crypts EDTA	Corrupted crypts DMH	Corrupted crypts EDTA
No. rats	25	10	25	10
Sum	331	570	202	1
Mean	13,24	57	8,08	0,10
Max	50	98	26	1
Min	0	30	1	0

#### II) Frequency of CCCF per crypts with fission in DMH-treated, and EDTA-treated rats

Table 1 indicates that 202 CCCF (37.9%) were recorded out of 533 crypts with fission in DMH-treated rats, and only one CCCF (0.1%) out of 571 crypts with fission in EDTA-treated rats. The difference in numbers of CCCF/crypts with fission between DMH-treated rats and EDTA-treated rats was significant (p<0.05).

**Fig 5:**
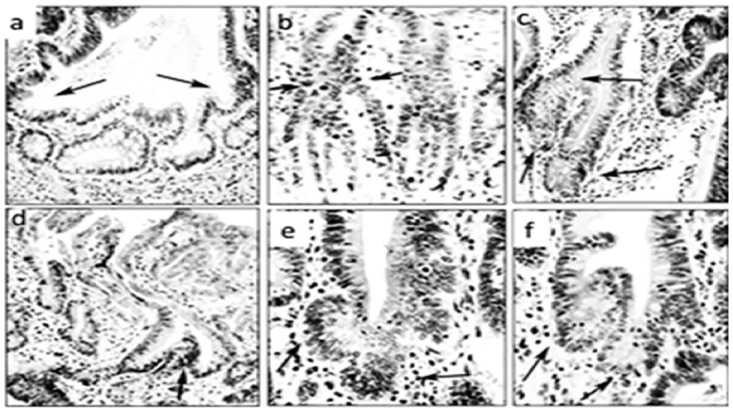
Corrupted colonic crypts showing top-down replacement by dysplastic epithelium from a conventional adenoma on top. **a**: Note non-dysplastic corrupted crypts underneath arrows, **b**: Two corrupted crypts with high grade dysplasia, the one on the left with additional asymmetric lateral fission (at arrows), **c-f**: Corrupted colonic crypts lined with dysplastic epithelium, **d**: Three-arm crypt, at arrow. (DMH-treated rats, hematoxylin-eosin, **a, b**: x10, **c, d** x10, **e, f**: x40).

The four polyps included in the Swiss roll sections were conventional adenomas, two of them with high-grade dysplasia (Fig 5). The basal aspect in these adenomas exhibited corrupted crypts, lined either with non-dysplastic (indigenous) epithelium or with dysplastic epithelium (Fig 5).

## Discussion

The results of the present survey showed that DMH-treated SD rats had a significantly higher number of CCCF than EDTA-treated SD rats. These findings strongly suggest that the carcinogen used in the experiment was crucial for the induction of CCCF in SD rats.

The CCCF described here differs from the appearance of aberrant crypt foci (ACF) in carcinogen-treated rodents reported by Bird *et al*. [14] inasmuch as the latter was detected following staining with methylene blue of the surface of the colonic mucosa. Thus, Bird *et al* assessed the ACF pattern by observing the mucosa “from the top” [14] and not, from the “side” in well-oriented histological sections, as in the present study.

According to Ghirardi *et al*. [16] and Ochiai *et al*. [17], the ACF phenomenon is infrequently found in the distal colon, the site of predilection for adenomas and carcinomas in carcinogen-treated rats. At variance with ACF, we found that CCCF predominantly evolving in the mid-distal colon, the site where neoplasias often develop in carcinogen-treated rats.

Recently Tan *et al*. found asymmetrical crypt budding in the colon of C57BL/6 mice [22]. It should be mentioned that in order to isolate colonic crypts from the *lamina propria*, the *muscularis mucosae* and the submucosa, the authors used an invasive method; colons were kept and rinsed on ice, incubated in sodium hypochlorite (5 minutes), in EDTA (90 minutes), transferred to PBS (20°C), shaken vigorous (5 times), and centrifuged (34g, 5 minutes). With this *“ex-vivo”* procedure, the authors found asymetic crypt buddings in untreated mice. Tan *et al* method [22] differs from the method described in this communication, inasmuch as the colons were fixed immediately after sacrifice, in formaldehyde. The crypts were subsequently observed “in situ”, in conventionally stained sections, a method less amenable to develop artifacts.

During crypt renewal, the stem cells at the crypt bottom generate amplifying daughter cells that proliferate and differentiate while migrating upwards [21, 23, 24]. Wnt signaling is high at the bottom (where SCs reside) and low at the top. In contrast, the adenomatous polyposis coli (Apc) gene concentration is low at the crypt bottom and high at the top (the domain of differentiated cells) [21, 23, 24]. The Apc gene normally down-regulates Wnt signalling. Hence, Wnt and Apc gradients are important in crypt formation and regulation. Since both Apc and Wnt signalling components are required for mitosis, a zone emerges in the lower crypt where conditions are optimal for maximal cell division and mitosis orientation resulting in symmetric crypt fission [21, 23, 24].

In 1985, St Clair and Osborne isolated colonic crypts from young rats by the aid of a microdissection technique [25]. Crypts were scored as ordinary or in fission. The percentage of crypts in fission (PCF) reached peak values of 52% in the colon at 21-day post-parturition. From this time-onwards, the PCF dropped until the adult value of approximately 7% was reached. During this same period, the number of crypts increased from 2.2 X 10 to 6.5 X 10 in the colon. Thus, an inverse relationship was found between the percentage of crypts in fission and the number of crypts. Distribution of fissure heights in fission crypts did not change as the animal aged. The majority of the fissures were found in the lower 1/4 of the fission crypts, suggesting that as soon as the fissure extends beyond the stem cell zone, division into two crypts soon occurs [24]. More recently, Boman and Fields [21] found that APC-mutation-induced changes in the counter-current-like mechanism, triggering expansion of proliferative populations, driving crypt fission premalignant changes and adenoma development. This proliferative shift in normal-appearing crypts, not yet dysplastic, was the earliest-known biological alteration. Notably, crypts exhibiting this proliferative abnormality (e.g., FAP crypts) did not show any microscopically visible changes at histology. According to Boman and Fields [21] mutations in the APC gene, found in most colorectal cancers, would cause abnormal crypt production, disorientation of the crypts, and increased crypt production leading to colorectal adenomas. These authors [21] claimed that the crypts began to show abnormalities in histology only when they became dysplastic, i.e. during the formation of premalignant adenomas, which had a second hit at the APC locus. In present work, we found in addition, that colonic crypts of carcinogen-treated SD rats may also portray histological abnormalities in the absence of dysplastic cell features.

In conclusion, it was demonstrated that CCCF without dysplasia develop in carcinogen-treated SD rats. As judged by the figures presented, the possibility that the epithelium in those corrupted crypts was successively replaced by top-down growing dysplastic cells, could not be totally rejected. This is the first report showing that non-dysplastic CCCF may antedate the very early stages of colonic carcinogenesis in SD rats.
